# New Work by Dr. John Bell

**Published:** 1853-01

**Authors:** 


*New Work by Dr. John Bell.*—We perceive, by our exchanges, that Dr. John Bell, of Philadelphia, is engaged in preparing a work on Mineral Waters, designed as a companion to his treatise on Baths. Dr. B. invites communications from those residing at, or near any of the celebrated watering places of the United States. The N. Y. Med. Gazette states, that "Details are desired touching their accessibility, facilities, and accommodations, which those concerned will find their interest to furnish, obliging themselves rather than the author."

